# Anti-Chikungunya Viral Activities of Aplysiatoxin-Related Compounds from the Marine Cyanobacterium *Trichodesmium erythraeum*

**DOI:** 10.3390/md12010115

**Published:** 2014-01-03

**Authors:** Deepak Kumar Gupta, Parveen Kaur, See Ting Leong, Lik Tong Tan, Michèle R. Prinsep, Justin Jang Hann Chu

**Affiliations:** 1 Natural Sciences and Science Education, National Institute of Education, Nanyang Technological University, 1 Nanyang Walk, Singapore 637616, Singapore; E-Mails: deepak.k.july@gmail.com (D.K.G.); leongst1@gis.a-star.edu.sg (S.T.L.); 2 Laboratory of Molecular RNA Virology and Antiviral Strategies, Department of Microbiology, Yong Lin School of Medicine, NUHS, National University of Singapore, MD4 Level 5, Science Drive 2, Singapore 117597, Singapore; E-Mail: parveenk@nus.edu.sg; 3 Department of Chemistry, University of Waikato, Private Bag 3105, Hamilton 3240, New Zealand; E-Mail: michele@waikato.ac.nz

**Keywords:** marine cyanobacterium, *Trichodesmium erythraeum*, aplysiatoxin, antiviral, Chikungunya virus

## Abstract

Tropical filamentous marine cyanobacteria have emerged as a viable source of novel bioactive natural products for drug discovery and development. In the present study, aplysiatoxin (**1**), debromoaplysiatoxin (**2**) and anhydrodebromoaplysiatoxin (**3**), as well as two new analogues, 3-methoxyaplysiatoxin (**4**) and 3-methoxydebromoaplysiatoxin (**5**), are reported for the first time from the marine cyanobacterium *Trichodesmium erythraeum*. The identification of the bloom-forming cyanobacterial strain was confirmed based on phylogenetic analysis of its 16S rRNA sequences. Structural determination of the new analogues was achieved by extensive NMR spectroscopic analysis and comparison with NMR spectral data of known compounds. In addition, the antiviral activities of these marine toxins were assessed using Chikungunya virus (CHIKV)-infected cells. Post-treatment experiments using the debrominated analogues, namely compounds **2**, **3** and **5**, displayed dose-dependent inhibition of CHIKV when tested at concentrations ranging from 0.1 µM to 10.0 µM. Furthermore, debromoaplysiatoxin (**2**) and 3-methoxydebromoaplysiatoxin (**5**) exhibited significant anti-CHIKV activities with EC_50_ values of 1.3 μM and 2.7 μM, respectively, and selectivity indices of 10.9 and 9.2, respectively.

## 1. Introduction

Prokaryotic filamentous marine cyanobacteria have emerged as an important source of structurally novel natural products in drug discovery efforts [1,2,3]. To date, more than 450 compounds have been reported with a majority belonging to either the non-ribosomal peptide synthetase (NRPS) or hybrid polyketide synthase (PKS)-NRPS class [1,2,3]. A number of these compounds possess potent biological activities with specific drug targets, including Class 1 histone deacetylase (e.g., largazole), proteasome (e.g., carmaphycins A and B), voltage-gated sodium channels (e.g., kalkitoxin and hoiamide A), and falcipains (e.g., symplostatin 4). Due to their specific interactions with these molecular targets, marine cyanobacterial compounds are an attractive source of lead compounds for drug development, particularly in the area of cancer, neurological disorders, and infectious diseases [4].

Aplysiatoxin and related analogues, e.g., oscillatoxins and nhatrangins, are distinct polyketide classes of marine toxins isolated from several cyanobacterial species, including *Lyngbya majuscula*, *Schizothrix calcicola*, and *Oscillatoria nigro-viridis* [5,6,7]. Several aplysiatoxin-related analogues are known to possess potent tumor-promoting properties through the activation of protein kinase C (PKC) [8]. PKC belongs to a family of serine/threonine kinases and plays important roles in cell proliferation, differentiation and apoptosis [9]. Due to its importance in cellular signal transduction pathways, PKC is a potential drug target for the treatment of various diseases, particularly in cancer therapy [10]. Researches from Japan recently developed new simplified analogues based on aplysiatoxin class of molecules, e.g., aplog-1, with potent anti-proliferative properties as potential anticancer agents [11,12,13,14,15,16,17,18,19]. These synthetic analogues have been shown to inhibit the action of tumor promotors as well as prevent growth of cancer cells in similar ways to bryostatin 1 [11].

Chikungunya virus (CHIKV) is a mosquito-transmitted *Alphavirus* that has re-emerged as a significant public health threat in recent years [20]. CHIKV is the causative agent of chikungunya fever, which is characterized by myalgia, arthralgia, headaches, nausea, fever, and maculopapular rash [21]. In addition, recurrent myalgia and arthralgia have been known to persist for years after the infection clears in some patients [22,23]. The widening geographical distribution of the mosquito vectors, *Aedes albopictus* and *Aedes aegypti* has also led to researchers suggesting the possibility of CHIKV being established almost globally [24]. Despite this, there is currently no antiviral treatment or vaccine against CHIKV [25].

**Figure 1:**
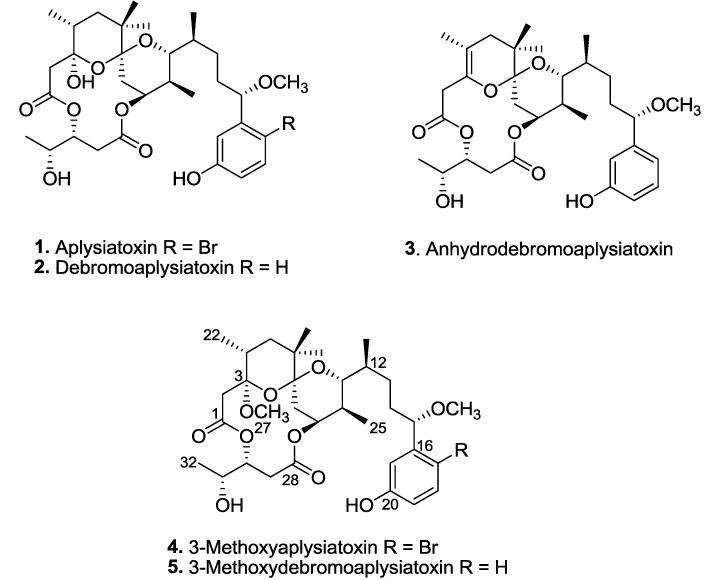
Aplysiatoxin-related compounds from *Trichodesmium erythraeum*.

Three known aplysiatoxins (**1**–**3**), as well as new analogues, compounds **4** and **5**, were isolated from the bioactive organic extracts of *Trichodesmium erythraeum* (TLT/PSK/001), recently collected from Pulau Seringat Kias, Singapore (Figure 1). Herein, we describe the structure elucidations of two new aplysiatoxin analogues, 3-methoxyaplysiatoxin (**4**) and 3-methoxydebromoaplysiatoxin (**5**), and report anti-CHIKV activities for compounds **2**, **3**, and **5**.

## 2. Results and Discussion

Phylogenetic analysis based on the 16S rRNA gene sequences identified the marine cyanobacterium as *Trichodesmium erythraeum*. Subsequent chemical investigation on the organic extract of *Trichodesmium erythraeum* (TLT/PSK/001) resulted in the isolation of known compounds, aplysiatoxin (**1**), debromoaplysiatoxin (**2**) and anhydrodebromoaplysiatoxin (**3**), as well as two new analogues, 3-methoxyaplysiatoxin (**4**) and 3-methoxydebromoaplysiatoxin (**5**) (Figure 1). The new aplysiatoxin analogues were isolated as colorless amorphous powder.

3-Methoxyaplysiatoxin (**4**) possesses a molecular formula of C_33_H_49_O_10_Br determined from HRESIMS due to a [M + Na]^+^ ion peak at *m/z* 707.2388 and accounts for nine degrees of unsaturation. The ^1^H NMR spectrum showed the presence of a trisubstituted benzene system with aromatic proton signals at δ 7.35, 6.99 and 6.65, two singlet methoxyl protons (δ 3.25 and 3.27), four doublet methyl protons (δ 0.79, 0.81, 0.83 and 1.19) as well as two singlet methyl protons (δ 0.93 and 0.94) (Table 1). In addition, ^13^C NMR and DEPT spectra showed 33 carbon signals, including two carbonyl signals at δ 170.17 (C-1) and δ 170.42 (C-28); two acetal carbons at δ 99.81 (C-3) and δ 102.07 (C-7); two methoxyl carbons at δ 49.40 (C-27) and δ 57.23 (C-33) attached to asymmetric carbons at δ 99.81 and δ 82.89 (C-3 and C-15, respectively); six methyl carbons at δ 16.06 (C-22), 29.08 (C-23), 27.44 (C-24), 14.00 (C-25), 11.94 (C-26) and 19.42 (C-32); six methylene carbons at δ 45.65 (C-2), 40.36 (C-5), 30.72 (C-8), 36.22 (C-13), 36.76 (C-14) and 32.46 (C-29); and twelve methine carbons at δ 34.19 (C-4), 74.67 (C-9), 36.91 (C-10), 72.49 (C-11), 33.93 (C-12), 82.89 (C-15), 133.75 (C-18), 115.52 (C-19), 116.80 (C-21), 73.09 (C-30) and 69.15 (C-31) (Table 1).

**Table 1:** 1D NMR spectral data for 3-methoxyaplysiatoxin (**4**) and 3-methoxydebromoaplysiatoxin (**5**) measured at 400 MHz (^1^H) and 100 MHz (^13^C) in CDCl_3_.

	3-Methoxyaplysiatoxin (4)			3-Methoxydebromoaplysiatoxin (5)	
Position	δ_H_ mult (*J* in Hz)	δ_C_		δ_H_ mult (*J* in Hz)	δ_C_
1		170.17 s			170.24 s
2	2.84 m	45.65 t		2.82 m	45.65 t
3		99.81 s			99.70 s
4	1.63 m	34.19 d		1.69 m	34.04 d
5	1.53 m	40.36 t		1.52 m	40.26 t
6		36.95 s			36.88 s
7		102.07 s			102.02 s
8	1.25 m	30.72 t		1.38 m	31.43 t
9	4.78 d (2.8)	74.67 d		4.76 d (2.5)	74.63 d
10	2.31 m	36.91 d		2.30 m	36.84 d
11	3.87 m	72.49 d		3.79 m	72.34 d
12	1.54 m	33.93 d		1.54 m	33.71 d
13	2.72 m	36.22 t		2.76 m	35.98 t
14	1.83 m	34.76 t		1.95 m	35.92 t
15	4.58 t (6.4)	82.89 d		4.06 t (6.9)	85.31 d
16		142.88 s			144.29 s
17		114.02 s		6.85 d (7.5)	119.10 d
18	7.35 d (8.8)	133.75 d		7.19 t (7.8)	129.86 d
19	6.65 dd (3.0, 8.6)	115.52 d		6.75 dd (1.5, 8.0)	115.02 d
20		156.05 s			156.55 s
21	6.99 d (2.8)	116.80 d		6.91 s	114.30 d
22	0.83 d (3.2)	16.06 q		0.86 d (6.7)	16.00 q
23	0.94 s	29.08 q		0.94 s	29.13 q
24	0.93 s	27.44 q		0.93 s	27.40 q
25	0.79 d (3.7)	14.00 q		0.72 d (1.2)	13.77 q
26	0.81 d (4.6)	11.94 q		0.75 d (5.2)	12.81 q
27 *O*Me	3.27 s	49.40 q		3.27 s	49.28 q
28		170.42 s			170.60 s
29	2.31 m	32.46 t		2.31 m	32.35 t
30	5.42 ddd (2.4, 4.0, 12.4)	73.09 d		5.43 ddd (2.5, 4.5, 10.8)	73.24 d
31	3.82 m	69.15 d		3.82 m	68.81 d
32	1.19 d (6.4)	19.42 q		1.19 d (6.4)	19.11 q
33 *O*Me	3.25 s	57.23 q		3.24 s	56.93 q

The second new analogue, 3-methoxydebromoaplysiatoxin (**5**), possesses a molecular formula of C_33_H_50_O_10_, as ascertained from HRESIMS based on the [M + Na]^+^ ion peak at *m/z* 629.3310, accounting nine degrees of unsaturation. Based on the 1D NMR spectra, **5** is structurally related to compound **4**. The ^1^H NMR spectrum indicated the presence of a meta-disubstituted benzene ring system with protons at δ 7.19, 6.91, 6.85 and 6.75. In addition, the ^1^H NMR spectrum showed two singlet methoxyl protons (δ 3.24 and 3.27), four doublet methyl protons (δ 0.72, 0.75, 0.86 and 1.19), and two singlet methyl protons (δ 0.93 and 0.94) (Table 1). The ^13^C NMR and DEPT spectra established the presence of 33 carbon signals, including two carboxylic ester carbons at δ 170.24 (C-1) and δ 170.60 (C-28); two acetal carbons at δ 99.70 (C-3) and 102.02 (C-7); six olefinic carbons (assigned to a *meta*-hydroxy substituted benzene ring); two methoxyl carbons at δ 49.28 (C-27) and 56.93 (C-33) attached to asymmetric carbons at δ 99.70 (C-3) and 85.31 (C-15), respectively; six methyl carbons at δ 16.00 (C-22), 29.13 (C-23), 27.40 (C-24), 13.77 (C-25), 12.81 (C-26) and 19.11 (C-32); six methylene carbons at δ 45.65 (C-2), 40.26 (C-5), 31.43 (C-8), 35.98 (C-13), 35.92 (C-14) and 32.35 (C-29); and twelve methine carbons at δ 34.04 (C-4), 74.63 (C-9), 36.84 (C-10), 72.34 (C-11), 33.71 (C-12), 85.31 (C-15), 119.10 (C-17), 129.86 (C-18), 115.02 (C-19), 114.30 (C-21), 73.24 (C-30) and 68.81 (C-31) (Table 1).

Extensive 1D and 2D NMR spectroscopic analysis of compounds **4** and **5** as well as comparison with NMR spectral data of known compounds revealed structural similarities to aplysiatoxin (**1**) and debromoaplysiatoxin (**2**), respectively. The presence of an additional methoxy group in either compound **4** or **5** was observed in the ^1^H and ^13^C NMR spectra, namely δ 3.27/3.27 (H-27) and δ 49.40/49.28 (C-27), respectively. In addition, the placement of the methoxy group at C-3 was revealed by a distinct correlation from H-27 to C-3 in the HMBC spectra of compounds **4** and **5**. Due to the structural similarities with aplysiatoxin and debromoaplysiatoxin, the absolute stereochemistry of the new methoxy analogues is assumed to be identical. The new methoxy analogues of aplysiatoxin and debromoaplysiatoxin are likely to be natural products and not artefacts. This is due to the relative amounts of **4** and **5** compared with **1** and **2**, respectively. In addition, it has been reported that aplysiatoxin is chemically unstable due to the labile hemiacetal hydroxyl group at C-3 and is easily eliminated to form compound **3** under weak acidic conditions [6]. Under such acidic conditions, it is unlikely that the tertiary carbocation formed at C-3 is attacked by solvent MeOH to form the methoxy functionality. Furthermore, steric hindrance by neighboring carbons could prevent the entry of MeOH to form a methoxy group at C-3.

**Figure 2:**
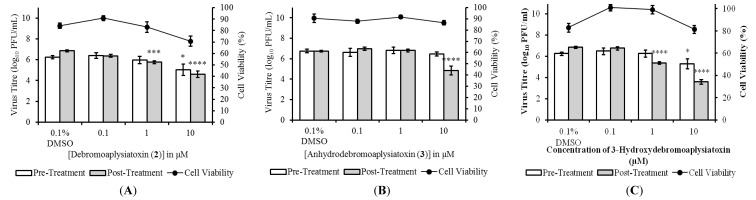
Dose-dependent studies of antiviral activities of (**A**) debromoaplysiatoxin (**2**); (**B**) anhydrodebromoaplysiatoxin (**3**); and (**C**) 3-methoxydebromoaplysiatoxin (**5**). SJCRH30 cells were treated with marine toxins either before (pre-treatment) or after (post-treatment) infection with CHIKV. Cell culture supernatants were harvested at 24 hpi (h postinfection) for titration via plaque assays. All compounds displayed statistically significant dose-dependent inhibition of CHIKV titres upon post-treatment and minimal cytotoxicity was observed upon treatment of SJCRH30 cells with marine compounds for 24 h. Statistical significance was analyzed by one-way ANOVA and Dunnett’s post-test (*, *p* ≤ 0.05; ***, *p* < 0.001; ****, *p* < 0.0001). (Error bars for dose-dependent and cell viability studies represent standard errors of means from three independent and from at least two independent experiments, respectively.)

The anti-CHIKV activities of compounds **1** to **5** were evaluated in both pre- and post-treatment studies. Pre-treatment studies were performed to determine the effects of the compounds on CHIKV binding and entry processes. In pre-treatment studies, BHK21 cells were treated with the compounds before being infected with CHIKV. Cell culture supernatants were harvested at 24 hpi (hours post-infection) for determination of viral titres via plaque assays. The effects of the marine-derived compounds on CHIKV replication within infected cells were assessed in post-treatment studies. For post-treatment studies, CHIKV-infected cells were treated with the compounds before cell culture supernatants were collected for virus titration at 24 hpi. Three compounds, **2**, **3** and **5**, displayed significant dose-dependent inhibition of CHIKV titres upon post-treatment (Figure 2), while compounds **1** and **4** did not result in any significant inhibition at 10 μM. Post-treatment resulted in statistically significant reduction in infectious CHIKV titre at treatment concentrations of 1 μM onwards for compounds **2** and **5**, and a concentration of 10 μM for compound **3**. Compound **2** displayed the most potent antiviral activity upon post-treatment with an EC_50_ value of 1.3 μM and a selectivity index (SI = CC_50_/EC_50_) of 10.9 (Table 2). The EC_50_ values of compounds **3** and **5** were 22.3 μM (SI of 11.4) and 2.7 μM (SI of 9.2), respectively (Table 2).

**Table 2:** Anti-Chikungunya viral activities of debromoaplysiatoxin (**2**), anhydrodebromoaplysiatoxin (**3**), and 3-methoxydebromoaplysiatoxin (**5**).

Compounds	EC_50_ (μM)	CC_50_ (μM)	SI (CC_50_/EC_50_)
Debromoaplysiatoxin (**2**)	1.3	13.9	10.9
Anhydrodebromoaplysiatoxin (**3**)	22.3	255.5	11.4
3-Methoxydebromoaplysiatoxin (**5**)	2.7	24.8	9.2

Pre-treatment of cells with compounds **1** to **5** prior to CHIKV infection did not result in any significant inhibition, except at a high concentration of 10 μM for compounds **2** and **5** (Figure 2). The minimal inhibition observed may have been due to the high treatment concentrations, which allowed the compounds to persist at sufficient levels in the cells to cause inhibition even after the pre-treatment period. Taken together, results from the pre-treatment and post-treatment studies suggested that the antiviral mechanism of compounds **2**, **3** and **5** is likely to target a step in the CHIKV replication cycle that occurs after viral entry. To ensure that the decreases in infectious titres were not due to cell cytotoxicity upon compound treatment, cell viability assays were performed using alamarBlue^®^ cell viability assay. Cell viability remained above 70% for all concentrations of the compounds assayed indicating minimal cytotoxicity (Figure 2).

This study represents the first report of antiviral activities of the aplysiatoxin-class of molecules. Debromoaplysiatoxin (**2**) and 3-methoxydebromoaplysiatoxin (**5**) were particularly active. Based on the activity profile, it is interesting to note that the absence of the bromine atom in **2** and **5** enhanced their antiviral properties. In addition, these compounds displayed anti-CHIKV effects at concentrations that resulted in minimal cytotoxicity. Debromoaplysiatoxin (**2**) and 3-methoxydebromoaplysiatoxin (**5**) may therefore represent a novel class of antivirals and further investigations into essential pharmacophores may allow generation of modified leads with improved pharmacological properties. Further studies in suitable cellular and murine models are warranted to determine their antiviral mechanism, as well as potential as therapeutics against CHIKV.

## 3. Experimental Section

### 3.1. General Experimental Procedures

A NIKON Eclipse TS100/TS100F inverted microscope was used for morphological characterization of the marine cyanobacterial samples. Proton, ^13^C, and 2D NMR spectra were recorded in CDCl_3_ on a 400 MHz Bruker NMR spectrometer using the residual solvent signals (δ_H_ at 7.26 ppm and δ_C_ at 77.36 ppm) as internal standards. Chemical shifts are reported in ppm and coupling constants (*J*) are reported in Hz. HRESIMS data were obtained using a Bruker Daltonics MicrOTOF™ series 89 mass spectrometer equipped with an ESI multimode ion source detector. HPLC isolation and purification of compounds **1** to **5** were conducted on a Shimadzu LC-8A preparative LC coupled to a Shimadzu SPD-M10A VP diode array detector. Optical rotations and UV spectra were measured on a Bellingham Stanley ADP 440 polarimeter and a Varian Cary 50 UV visible spectrophotometer, respectively.

### 3.2. Marine Cyanobacterial Collection

About 1.0 L of the benthic filamentous marine cyanobacterium, *Trichodesmium erythraeum*, were collected from the C-shaped manmade lagoon (N 1°13′43.56′′, E 103°51′14.69′′) at Pulau Seringat Kias, Singapore, during low tides on 7 August 2012. Small amounts of cyanobacterial samples were rinsed off with distilled water before examination under microscope (10× magnification) for morphological characterization. Remaining samples were stored in 70% aqueous ethanol at −20 °C, before chemical workup. A voucher specimen of this microalga is maintained at NIE under the code TLT/PSK/001. Additional cyanobacterial samples were preserved in 10 mL Nalgene tubes containing RNAlater^®^ solution (Life Technologies Corporation, Carlsbad, CA, USA) for subsequent DNA extraction.

### 3.3. DNA Extraction and PCR Amplification of 16S rRNA

Marine cyanobacterial genomic DNA was extracted using PowerSoil^®^ DNA Isolation Kit (MO BIO Laboratories, Inc., Carlsbad, CA, USA) following the manufacturer’s specifications. Concentration of extracted cyanobacterial DNA and purity were measured on a NanoDrop 1000 spectrophotometer (Thermo Fisher Scientific Inc., Waltham, MA, USA). The 16S rRNA gene was PCR amplified from the isolated genomic DNA using specific cyanobacterial primers 27F 5′-AGAGTTTGATCCTGGCTCAG-3′ and 809R 5′-GCTTCGGCACGGCTCGGGTCGATA-3′. The PCR reaction contained 1.0 µL (~100 ng) of DNA, 10 µL of 10× AmpliTaq Gold reaction buffer (Life Technologies Corporation, Carlsbad, CA, USA), 2 µL of 10 mM dNTP mix, 2 µL of 10 µM of each primer, 3 µL of 100 mM magnesium chloride, 10 µL of AmpliTaq Gold DNA Polymerase (5 U/µL), and 70 µL of nuclease-free water for a total volume of 100 µL. The PCR reactions were performed using the GeneAmp^®^ 9700 PCR System (Life Technologies Corporation, Carlsbad, CA, USA) with the following cycling conditions: initial denaturation for 5 min at 95 °C, amplification by 35 cycles of 1 min at 95 °C, 1 min at 58 °C and 1 min at 72 °C, and final elongation for 7 min at 72 °C. PCR amplified products were purified using QIAquick PCR Purification Kit and QIAquick Gel Extraction Kit (QIAGEN, Valencia, CA, USA) following the manufacturer’s specifications.

### 3.4. Gene Sequencing and Phylogenetic Analysis

All purified PCR products of 27F and 809F primers were modified and sequenced by using the M13 forward primer 5′-TGTAAAACGACGGCCAGT-3′ and M13 reverse primer 5′-CAGGAAACAGCTATGACC-3′ (1st BASE, Singapore) and tailing the 5′ end in order to allow direct sequencing of 16S rRNA gene by the BigDye^®^ Terminator v3.1 Cycle Sequencing Kit and an ABI 3730 DNA Analyzer (Applied Biosystems, Carlsbad, CA, USA). Sequencing reactions and cleanup were performed according to 1st BASE instructions. Sequences were aligned using online analysis tools, SEQ MATCH, from Ribosomal Database Project (RDP) with the following set of parameters strain (Both), Source (Isolates), Size (≥1200) and Quality (Good). Phylogeny analysis was carried out using the TREE BUILDER tool.

### 3.5. Extraction and Isolation of Compounds **1** to **5**

The thawed marine cyanobacterial sample (~142 g dry wt.) was exhaustively extracted using MeOH/CHCl_3_ (1:2, v/v) to produce an organic extract which was filtered through cheese cloth and the solvents removed *in vacuo* to yield a dark green extract (4.54 g). The extract was then subjected to vacuum flash chromatography (VFC), normal phase silica gel (0.063–0.200 mm, 70–230 mesh ASTM, Merck, Darmstadt, Germany), using a stepwise gradient solvent system of hexanes, EtOAc, and MeOH in increasing polarity to provide nine fractions. The eluents were concentrated *in* *vacuo* before storage in 4 dram (15 mL) vials. All fractions were tested at 100 and 10 ppm, in duplicate, using the brine shrimp (*Artemia salina*) toxicity assay [26]. The fraction eluted with hexanes/EtOAc (1:4) gave 95% and 60% toxicity in the brine shrimp toxicity assay when tested at 100 and 10 ppm, respectively. This bioactive fraction was further fractionated through a SEP-PAK RP-18 cartridge (10 g/70 mL SPE Isolute column) using a combination of MeOH and H_2_O into three sub-fractions. The brine shrimp active sub-fraction eluted with 10% aqueous MeOH was subjected to further purification by Preparative C_18_ RP-HPLC [Phenomenex Sphereclone 5 µm ODS, 250 × 10.00 mm, CH_3_CN/H_2_O (62:38, v/v) at 2.8 mL/min, UV detection at 254, 230 and 210 nm] to obtain anhydrodebromoaplysiatoxin (**3**, 2.8 mg, *t*_R_ = 19.4 min), debromoaplysiatoxin (**2**, 3.1 mg, *t*_R_ = 20.6 min), 3-methoxydebromoaplysiatoxin (**5**, 3.4 mg, *t*_R_ = 22.0 min), 3-methoxyaplysiatoxin (**4**, 1.6 mg, *t*_R_ = 37.2 min) and aplysiatoxin (**1**, 1.9 mg, *t*_R_ = 39.1 min).

3-Methoxyaplysiatoxin (**4**): white, amorphous solid; [α] ^25^_D_ −17.8 (c 0.5, MeOH); UV (MeOH) λ_max_ 213 nm (ε 5420); ^1^H NMR (400.13 MHz, CDCl_3_) and ^13^C NMR (100.62 MHz, CDCl_3_) spectral data see Table 1; HRESIMS *m/z* [M + Na]^+^ 707.2388 and 709.2276 (calcd. for C_33_H_49_O_10_^79^BrNa, 707.2401 and for C_33_H_49_O_10_^81^BrNa, 709.2388).

3-Methoxydebromoaplysiatoxin (**5**): white, amorphous solid; [α] ^25^_D_ −21.7 (c 0.5, MeOH); UV (MeOH) λ_max_ 223 nm (ε 4660); ^1^H NMR (400.13 MHz, CDCl_3_) and ^13^C NMR (100.62 MHz, CDCl_3_) spectral data see Table 1; HRESIMS *m/z* [M + Na]^+^ 629.3310 (calcd. for C_33_H_50_O_10_Na, 629.3296).

### 3.6. Anti-CHIKV Activities of Compounds **1** to **5**

#### 3.6.1. Cell Lines and Compound Preparations

BHK21 (baby hamster kidney) cells and SJCRH30 rhabdomysarcoma cells (ATCC CRL-2061) were maintained in RPMI-1640 medium supplemented with 10% fetal calf serum (FCS) at 37 °C and 5% CO_2_. C6/36 mosquito cells isolated from *Aedes albopictus* embryonic tissue were cultured in L-15 medium containing 10% FCS at 28 °C. CHIKV-122508 (SGEHICHD 122508, kindly provided by the Environmental Health Institute, Singapore) was propagated in C6/36 cells and stored at −80 °C prior to use. Compounds **1** to **5** were each dissolved in DMSO to a final stock concentration of 10 mM and stored at −80 °C.

#### 3.6.2. Cell Viability Assay

The cytotoxicity profiles of compounds **1** to **5** were assessed by alamarBlue cell viability assay (Invitrogen). In brief, SJCRH30 were seeded in 96-well plates at a density of 1.5 × 10^4^ cells per well and incubated overnight at 37 °C with 5% CO_2_. Compounds were diluted in RPMI with 2% FCS prior to being incubated with SJCRH30 cell monolayers for 24 h at 37 °C with 5% CO_2_. After the incubation period, the compounds were removed and cells were washed once with PBS (phosphate buffered saline) before being incubated with alamarBlue^®^ reagent diluted in RPMI with 2% FCS at a dilution ratio of 1:10. Fluorescence was detected at an excitation wavelength of 570 nm and an emission wavelength of 585 nm with the Infinite 200 Pro multiplate reader (Tecan, Männedorf, Switzerland). Measurements from cells treated with compounds or 0.1% DMSO solvent (control) were normalized against those from untreated cells. The 50% cytotoxic concentration (CC_50_) was determined as the treatment concentration that resulted in 50% cell viability.

#### 3.6.3. Dose-Dependent Anti-Chikungunya Assay

The anti-CHIKV activities of compounds **1** to **5** were investigated using dose-dependent drug treatments on SJCRH30 cells. SJCRH30 cells were seeded at a density of 1.5 × 10^4^ cells per well in 96-well plates and incubated overnight at 37 °C with 5% CO_2_. For both pre- and post-treatment assays, each compound was diluted in RPMI with 2% FCS. For pre-treatment assays, cells were treated with each compound for 2.5 h at 37 °C prior to being washed once with PBS. Treated cells were incubated with CHIKV-122508 at an MOI (multiplicity of infection) of 10 for 1.5 h to allow viral attachments to the cells. After the adsorption period, cell monolayers were washed twice with PBS before being incubated in RPMI with 2% FCS at 37 °C and 5% CO_2_. After 24 h, cell culture supernatants were harvested for titration using plaque assay. For post-treatment assays, SJCRH30 cells were infected with CHIKV-122508 at an MOI of 10 for 1.5 h prior to being washed twice with PBS. Infected monolayers were then incubated with varying concentrations of each compound for 24 h at 37 °C with 5% CO_2_ before supernatants were collected for plaque assay. The 50% effective concentration (EC_50_) was determined as the concentration that produced 50% of maximal inhibition of log_10_ virus titre when compared against the solvent control.

#### 3.6.4. Plaque Assay

For quantification of infectious virus titer, BHK21 cells were seeded onto 24-well plates and incubated overnight at 37 °C with 5% CO_2_. Supernatants from pre- and post-treatment assays were diluted in 10-fold dilutions in RPMI with 2% FCS before being used to infect the cells for 1.5 h at 37 °C. Infected monolayers were washed twice with PBS and overlaid with 1% carboxymethyl cellulose (CMC) in RPMI with 2% FCS. Plaque assay plates were incubated for 3 days at 37 °C with 5% CO2. After the incubation period, CMC was removed and cells were stained and fixed with 10% paraformaldehyde–1% crystal violet (Sigma-Aldrich Chemical, St. Louis, MO, USA) solution for visualization and counting of plaques. Virus titers were expressed as plaque-forming units (PFU) per milliliter.

#### 3.6.5. Statistical Analysis

Data from dose-dependent studies were evaluated for significance by one-way analysis of variance (ANOVA) tests. For samples that showed statistical significance (*p* < 0.05), a Dunnett’s post-test was performed to compare data from compound treatment against the solvent (0.1% DMSO) control in order to determine the compound concentrations which resulted in significant inhibition of viral titre.

## 4. Conclusions

Aplysiatoxin-related compounds, including two new analogues, 3-methoxyaplysiatoxin (**4**) and 3-methoxydebromoaplysiatoxin (**5**), have been reported for the first time from the marine cyanobacterium, *Trichodesmium erythraeum*. These compounds, particularly the debromo analogues **2** and **5**, showed significant anti-CHIVK activities in post-treatment of infected SJCRH30 cells with EC_50_ values of 1.3 and 2.7 μM, respectively. The mode of action of the antiviral compounds is likely to target a step in the CHIKV replication cycle that occurs after viral entry. Aplysiatoxins may therefore represent a novel class of antiviral agents and warrant further biological investigation into their mode of action.
